# Prader-Willi Syndrome and Growth Hormone Deficiency

**DOI:** 10.4274/jcrpe.1228

**Published:** 2014-06-05

**Authors:** Zehra Aycan, Veysel Nijat Baş

**Affiliations:** 1 Dr. Sami Ulus Women Health, Children’s Education and Research Hospital, Clinics of Pediatric Endocrinology, Ankara, Turkey; 2 Yıldırım Beyazıt University School of Medicine, Department of Pediatric Endocrinology, Ankara, Turkey; 3 Kayseri Training and Education Hospital, Department of Pediatric Endocrinology, Kayseri, Turkey

**Keywords:** Prader-Willi syndrome, Growth hormone deficiency, growth hormone treatment

## Abstract

Prader-Willi syndrome (PWS) is a rare multisystem genetic disorder demonstrating great variability with changing clinical features during patient’s life. It is characterized by severe hypotonia with poor sucking and feeding difficulties in early infancy, followed by excessive eating and gradual development of morbid obesity in later infancy or early childhood. The phenotype is most probably due to hypothalamic dysfunction which is also responsible for growth hormone (GH) and thyroid-stimulating hormone (TSH) deficiencies, central adrenal insufficiency and hypogonadism. The multidimensional problems of patients with PWS can be managed with multidisciplinary approach. Reduced GH secretion, low peak GH response to stimulation, decreased spontaneous GH secretion and low serum IGF-1 levels in PWS patients have been documented in many studies. GH therapy has multiple beneficial effects on growth and body composition, motor and mental development in PWS patients. The recommended dosage for GH is 0.5-1 mg/m2/day. GH therapy should not be started in the presence of obstructive sleep apnea syndrome, adenotonsillar hypertrophy, severe obesity and diabetes mellitus. GH treatment should be considered for patients with genetically confirmed PWS in conjunction with dietary, environmental and life-style measures.

## INTRODUCTION

Prader-Willi syndrome (PWS) was first defined in 1956 by Swiss scientists (1). The prevalence of the disease, which is similar in both genders, is between 1/10 000 and 1/30 000 with approximately 400 000 PWS patients living worldwide (2,3,4). In previous years, due to morbid obesity and related complications, no patient lived over the age of 50 years, but today, the life span of these patients has increased. PWS is characterized by a recognizable pattern of physical findings with significant cognitive, neurologic, endocrine and behavioral abnormalities caused by lack of expression of genes from an imprinted region of the paternally inherited chromosome 15q11-q13, near the centromere (5). These patients experience difficulty in feeding due to hypotonia in infancy leading to growth retardation, but they become obese after this period due to their uncontrollable craving for food. The degree of obesity increases with age and becomes permanent. Clinical presentation and physical characteristics of PWS show variations among age groups (2,3,4,5,6,7). Since obesity and related complications are important causes of morbidity and mortality in PWS cases, early diagnosis and careful follow-up are of great importance. 

Pediatric endocrinologists, using a holistic approach, need to manage the age-related worsening obesity and associated complications in addition to the central hypothyroidism due to hypothalamo-hypophyseal dysfunction, the central adrenal insufficiency, hypogonadotropic hypogonadism and growth hormone (GH) deficiency in PWS patients.

The present review will focus on the characteristic findings of patients with PWS, with special reference to GH and GH treatment.

**Clinical Findings**

The clinical signs of PWS vary by age group. 

**Prenatal period:** Decreased fetal activity, polyhydramnios, breech presentation and abnormal position of the hands and feet on ultrasonography in the 3rd trimester (elbows in flexion and feet in dorsi extension) constitute the characteristic findings of PWS in this period of life (7,8). 

**Infancy:** Severe hypotonia, difficulty in feeding due to poor sucking, growth retardation, weak crying, genital hypoplasia (cryptorchidism, scrotal and clitoral hypoplasia), dermal and ocular depigmentation are the main findings (3,4,6,8). 

**Early childhood:** Hyperphagia and obesity (unless food consumption is restricted externally), abnormal body composition (decreased muscular mass, increased fat mass), reduction in resting energy expenditure, short stature, behavior problems (eating-related problems such as hiding food, taking food out of the trash can and stealing food) are the main findings in this age group (3,4,6).

**Late childhood and adolescence:** Delayed puberty (descent of the testes into the scrotum may be delayed until adolescence in boys, menarche may be delayed sometimes to as late as age 30 years in girls), lack of pubertal height gain, morbid obesity and related complications [e.g. obstructive sleep apnea (OSA) syndrome, cor pulmonale, diabetes mellitus, atherosclerosis, hypertension], osteoporosis, scoliosis (30%), behavior problems, epilepsy (25%) are the main findings in this age group (2,3,7).

Since PWS is associated with a hypothalamo-hypophyseal function disorder, the patients should be investigated also for central hypothyroidism and hypophyseal adrenal insufficiency at the time of diagnosis. Mental and motor retardation are almost always present and IQ is generally 40 points below the mean values of the population. The diagnosis must certainly be verified by genetic testing in all patients suspected to have PWS. Appropriate follow-up and treatment approaches are important in the patients who are diagnosed genetically. In PWS, regulating the diet, programming exercise and management of endocrinological, neurological and psychological problems in every period of life require a multidisciplinary approach. 

**GH Deficiency**

Although the prevalence of GH deficiency in PWS patients changes according to the diagnostic criteria used, it has been reported to be present in 40% to 100% of patients (6,9,10). GH deficiency becomes prominent in the second decade of life in children that have not received GH replacement. These children fail to show the acceleration in height gain seen in puberty and the mean final height without treatment is 155 cm in boys and 148 cm in girls (2,4,9).

Since PWS is associated with hypothalamo-hypophyseal dysfunction, GH secretion is generally reduced in such cases. This is not related to obesity. Unlike obese individuals who show a decrease in GH secretion while maintaining normal insulin-like growth factor-1 (IGF-1) levels, in PWS patients, IGF-1 levels are also decreased (11,12). The criteria used for normal children can also be used for the diagnosis of growth retardation in these children. Of note, nutrition deficiency and presence of hypothyroidism particularly in infancy should be excluded, since these conditions would influence growth rate. GH deficiency should be considered in children with PWS who show an inadequate growth rate but have no nutritional deficiency or hypothyroidism. Twenty four-hour GH secretion is inadequate in 58%-100% of PWS patients (12,13,14,15). While a low growth rate in PWS is considered as an indicator of GH deficiency by some authors and provocative GH testing is not thought to be necessary (4,6,9), provocative tests for GH deficiency are generally recommended to be performed in PWS patients with inadequate growth rates as well as in those who show adequate growth. Depending on the results of the GH stimulation tests, GH treatment is recommended for patients with inadequate, as well as for those with adequate growth rates. The decision should be made together with the family after informing them about the benefits of GH treatment in detail. Moreover, the diagnosis of PWS needs to be verified by genetic testing before starting treatment with GH (9,12,15,16,17).

GH treatment in PWS was first applied in 1990s. Treatment outcomes and benefits for these patients were presented in the national conference of the Prader-Willi Syndrome Association which took place in 1998-1999 in the United States of America (18). The Federal Drug Association approved the use of GH in PWS in 2000 and the treatment has become widespread since. With the experiences gained in the subsequent years, attention has been drawn to the fact that starting treatment at a younger age improved its benefits, particularly its effect on muscle tonus. Nevertheless, no consensus could be reached on age of starting treatment (12,13,14,15,16). However, many researchers consider that starting treatment before the age of 2 years, at a time which coincides with onset of obesity, has a more beneficial effect on clinical improvement. However, this is not always possible, since in many countries, documentation of low growth rate is required to start GH treatment (9,12,16,17).

**Effects of GH Therapy **

GH has beneficial effects on linear growth, body composition, basal energy consumption, muscle strength, exercise tolerance, decrease in free fat mass, bone density, lipid profile and physical and cognitive functions (12,13,14,15,19,20,21,22,23,24). Age of starting treatment, treatment dose and duration of treatment are the determinants of therapy response (2,4,9). In a study that compared PWS patients receiving GH therapy with those who did not, it was reported that the group receiving treatment had a lower body fat, increased muscle mass, better lipid profile and better motor function. In that study, the age of starting treatment was 4-20 months and the authors suggested that early treatment might explain the significant differences observed (13,14,15). Many studies revealed that GH therapy provides significant improvement in body composition and height, particularly in the first year of treatment (6,9,12,13,14,15). In addition to height and body composition, GH has been reported to have beneficial effect on cognitive functions and behavioral patterns. It has been emphasized that significant improvement is observed in the mobility scores, motor skills, language and cognitive functions of the patients on the development tests performed before treatment and during follow-up (9,23,24). 

The results of the Bakker et al’s study (25), conducted on 60 PWS patients who began to receive GH therapy at the age of 3-7 years and were able to continue it for 8 years, are quite important to understand the effects of GH treatment. This study is unique as it comprises PWS patients who received GH therapy for the longest period. Whilst the height standard deviation (SD) of the patients was under -2 SD before treatment, all were reported to achieve normal height SD after treatment. Whilst lean body mass significantly increased and fat SD% significantly decreased in the first year of treatment, no significant difference was observed in the subsequent years and at the end of the 8-year treatment period versus baseline in either of these parameters. The authors attributed these results to the effect of GH therapy in hindering obesity. Nonetheless, BMI values of the cases remained +2 SD above that of normal children but were below that of non-treated children with PWS. Moreover, it was reported that with GH therapy, there was no increase in fasting insulin, homeostasis model assessment of insulin resistance and serum glucose level that no diabetes developed in any of the patients, but that a significant decrease was noted in serum cholesterol and LDL levels. 

In a study that compared the final heights in PWS patients who received GH with those who did not, final height was 171±8 cm in boys and 158±4 cm in girls in the treatment group, whereas it was 154±9 cm in boys and 144±6 cm in girls in the group without treatment (26).

**Pretreatment Evaluation**

Before starting GH therapy, one should perform anthropometric evaluation and systemic examination including height, height SD score (SDS), body weight, body mass index (BMI) SDS, waist/hip ratio, bone age and pubertal staging assessment. Moreover, central hypothyroidism and adrenal insufficiency should certainly be explored and L-thyroxine therapy should be started in case hypothyroidism is detected. Presence of adrenal insufficiency should be explored by estimation of serum adrenocorticotropic hormone (ACTH)-cortisol levels in blood samples obtained in the early morning and by low-dose ACTH stimulation test when needed (2,3,4,5,7,8,9,10). It has been reported that undetectable adrenal insufficiency might be responsible for mortality during GH therapy in PWS patients. It has also been reported that losing the patients particularly because of infection might be associated with non-use of steroid replacement therapy. Hydrocortisone therapy in stress doses is recommended in moderate and severe infections (27,28,29). In addition, evaluation of growth rate is needed and GH stimulation tests should be performed in those with as well as without inadequate growth rate. Serum IGF-1 and IGF binding protein-3 (IGFBP-3) levels are important parameters both before treatment and during monitoring. IGF-1 levels should be kept under +2 SD over the course of treatment period and/or IGF-1/IGFBP-3 ratio should not be allowed to exceed pretreatment level (2,9,12,13,14,15,23). Metabolic status should be evaluated if the patient is older than 12 years, or in the presence of morbid obesity. Fasting blood glucose, fasting insulin and lipid profiles should be assessed and oral glucose tolerance testing (OGTT) should be performed in the presence of additional risks (family history of diabetes or acanthosis nigricans, etc.). Obese patients should undergo AST, ALT testing and abdominal ultrasonography for hepatic steatosis and body composition should be evaluated (DEXA or bioelectrical impedance, if available) (4,7,9,30). The diagnosis of PWS must be verified genetically before treatment (2,4,7,9,10). Diet for PWS patients should be arranged under the supervision of a dietitian both before and over the course of treatment. Inadequate feeding due to hypotonia in infancy unfavorably influences the mental development of these patients. These patients must be fed via gastrostomy if necessary. The struggle against obesity in childhood should include regulation of diet and physical exercises and in conjunction with GH therapy. For developmental and cognitive evaluation, age-match psychomotor testing should be performed. The patients should be evaluated for motor function and directed for physical therapy, psychotherapy and occupational therapy if necessary (4,9,12,13,14,15,24,25). ENT consultation should be requested if breathing disorders during sleep, snoring, enlarged tonsils and adenoids are present. Tonsillectomy and adenoidectomy should be performed if indicated. Presence of sleep apnea should be assessed by polysomnography before starting GH therapy. It is necessary to perform polysomnographic evaluation at regular intervals over the course of monitoring in patients under treatment (2,4,7,9,10). Presence of scoliosis should be assessed by roentgen graphic imaging of the vertebrae and orthopedic consultation should be requested if indicated (19,20,31). The family should be informed in writing about the benefits and risks of GH therapy as well as those of careful monitoring. Permission should be obtained for follow-up and treatment from the legal representative of the patient or from the patient him/herself depending on the age and cognitive status of the patient (4,9).

**Efficacy of GH Dosage**

The best response to GH in PWS patient is observed in the first 12 months of treatment. Response to GH therapy changes according to age of starting treatment, degree of growth retardation and duration of treatment. Improvement in linear growth, bone density and body composition continues in those who receive therapy for 5 years. Nevertheless, it has been reported that long-term therapy may always not result in complete improvement in body composition, but stabilization is provided. Although early treatment is important for the improvement in body composition, generally, in practice, it is possible to start treatment only after age 2 years (9,12,13,14,15,19,20,21,22,23,24,32).

Treatment can be started in a dose of 0.034 mg/kg/day (0.24 mg/kg/week) in infants and toddlers. IGF-1 and IGFBP-3 levels are used to specify the dose of GH therapy. The dose can be increased up to 1 mg/m2/day so that IGF-1 levels are between the ranges +1 SD and +2 SD depending on age (2,7,9,33). The dose can be decreased if serum IGF-1 level increases over +2 SD. 

Benefits of continuing GH therapy in adulthood remains unclear. However, a moderate improvement has been observed in body composition and cognitive functions in PWS patients who received treatment in adulthood but not in childhood, suggesting that beneficial effects are still present even after the epiphyses are fused (26,33,34). Similar to the dose used for GH deficiency in adults, a daily dose of 0.1-0.2 mg is recommended; it should be adjusted so that the IGF-1 level is maintained between 0 SDS and - 2 SDS to reduce the probability of adverse events (7,9,26,33,34).

**Contraindications to GH Treatment**

Severe obesity, uncontrolled diabetes mellitus, untreated severe OSA, active cancer and psychosis are the accepted contraindications for GH therapy in PWS patients (2,4,7,9).

**Adverse Events and Safety**

As true for other patients receiving GH, patients with PWS should be monitored for potential adverse events by periodic complete blood count, thyroid function evaluation and evaluation of glucose metabolism. In addition, with the prediction that GH therapy will enhance adenotonsillar hypertrophy in PWS, the patients should be questioned for shortness of breath, snoring and sleep apnea and, if necessary, should be evaluated via polysomnography on the 3rd and 6th months after treatment. It should be kept in mind during monitoring that glucose metabolism disorders and type 2 diabetes are expected to be more common in PWS patients due to concomitant obesity. However, it has been reported that GH treatment showed no adverse effects on glucose parameters and lipid profile in PWS patients (26). Scoliosis and femur head epiphysis dislocation need to be explored at each control visit. Serum IGF-1 level should be kept under +2 SD and it is necessary to decrease the treatment dose in cases with higher levels. In patients with high serum IGF-1 levels, an effort should be made to keep the IGF-1/IGFBP-3 ratio at the same level as the baseline (9,26,34).

Worldwide, 28 deaths have been reported among PWS patients who had been receiving GH. Common characteristics of these patients were that they generally died due to respiratory problems or respiratory system infections in the first 9 months of treatment and that these patients were morbid obese (7,9,28,35). Although respiratory complications are seen in the first 3 months of GH therapy in patients with morbid obesity, these adverse events are also reported in the majority of the children. Indeed, mortality rate is reported to be high in PWS, but it has not been possible to establish a direct association between deaths and GH treatment. Moreover, no difference was found in mortality rates between the groups receiving treatment and those who were not. An increase in risk of sudden death has been reported in children with PWS, independent of GH therapy (28,35,36,37,38).

Whilst GH therapy leads to adenotonsillar hypertrophy and obstructive apnea on the one hand, on the other hand, it improves central hypoventilation via direct effect on hypothalamic function (9,19,20,21,32,35). Improvement was demonstrated in respiratory disorders and respiratory functions via this mechanism in cases with high IGF-1 levels (7,8,9,37). In view of all this information, it is recommended that treatment should be started after adenotonsillar surgery in children who have obstructive apnea before treatment; that steroid replacement therapy should be given in stress doses for infections that develop during treatment; that GH therapy should be stopped until the infection has subsided; and that GH therapy should be discontinued in the event of a new upper respiratory obstruction and/or sleep apnea which has developed after the onset of treatment for the first time (4,9).

The patient should be monitored periodically over the course of GH therapy. Polysomnography and ear-nose-throat examination should be performed on the 6th week, 3rd month and on the 6th month of treatment and whenever necessary (9,14). Fasting blood glucose, OGTT, IGF-1, free thyroxine and thyroid-stimulating hormone testing should be performed on the first month of treatment and/or whenever necessary and orthopedic examination and X-ray imaging of the spine should be performed in case of necessity (4,7,9,10,14,25).

In conclusion, GH treatment therapy appears to be of benefit in selected cases of PWS, provided that these patients are monitored carefully and precisely. Diet and physical activity should continue over the course of treatment. It will be reasonable to discontinue the treatment in the presence of infection and respiratory obstruction. Steroid replacement in stress doses needs to be given in case of infections. 
